# Evaluation of Three Automated Nucleic Acid Extraction Systems for Identification of Respiratory Viruses in Clinical Specimens by Multiplex Real-Time PCR

**DOI:** 10.1155/2014/430650

**Published:** 2014-04-28

**Authors:** Yoonjung Kim, Mi-Soon Han, Juwon Kim, Aerin Kwon, Kyung-A Lee

**Affiliations:** ^1^Department of Laboratory Medicine, Yonsei University College of Medicine, 211 Eonju-ro, Gangnam-gu, Seoul 135-720, Republic of Korea; ^2^Department of Laboratory Medicine, Yonsei University Wonju College of Medicine, Wonju, Republic of Korea; ^3^Green Cross Laboratories, Yongin, Republic of Korea

## Abstract

A total of 84 nasopharyngeal swab specimens were collected from 84 patients. Viral nucleic acid was extracted by three automated extraction systems: QIAcube (Qiagen, Germany), EZ1 Advanced XL (Qiagen), and MICROLAB Nimbus IVD (Hamilton, USA). Fourteen RNA viruses and two DNA viruses were detected using the Anyplex II RV16 Detection kit (Seegene, Republic of Korea). The EZ1 Advanced XL system demonstrated the best analytical sensitivity for all the three viral strains. The nucleic acids extracted by EZ1 Advanced XL showed higher positive rates for virus detection than the others. Meanwhile, the MICROLAB Nimbus IVD system was comprised of fully automated steps from nucleic extraction to PCR setup function that could reduce human errors. For the nucleic acids recovered from nasopharyngeal swab specimens, the QIAcube system showed the fewest false negative results and the best concordance rate, and it may be more suitable for detecting various viruses including RNA and DNA virus strains. Each system showed different sensitivity and specificity for detection of certain viral pathogens and demonstrated different characteristics such as turnaround time and sample capacity. Therefore, these factors should be considered when new nucleic acid extraction systems are introduced to the laboratory.

## 1. Introduction

Respiratory viruses can cause mild to severe illnesses as well as frequent complications. They frequently cause pneumonia in children, especially those younger than 2 years (up to approximately 80%) [1, 2]. For adult patients in the ICU, these pathogens account for 28% of pneumonia cases, with mortality rates comparable to those of bacterial pneumonia [3]. The mean annual incidence of respiratory tract infections in the United States was reported to be 4.2 and 1.2 for the first and the second years of a child's life, respectively. Furthermore, 27% of respiratory tract infections resulted from coinfection with two or more viruses, including rhinovirus, human coronavirus, and adenovirus [4]. Since the year 2000, many new respiratory viruses have been identified including H5N1 avian influenza, SARS-coronavirus, human coronavirus NL63, human coronavirus HKU1, human metapneumovirus, human bocavirus, and human rhinovirus type C [5, 6]. Therefore, simultaneous identification of multiple viruses is needed for timely patient management.

Detection or characterization of the respiratory viruses by conventional diagnostic techniques such as cell culture, direct fluorescent antibody detection (DFA), and serological testing can be difficult and time-consuming [7–9]. Thus, rapid and highly accurate PCR methods have been numerously evaluated and multiplex real-time (RT)-PCR method is currently considered as the best technique for the detection and typing of comprehensive panel for many common respiratory viruses [7, 10, 11].

High-quality nucleic acid extraction is necessary for the multiplex RT-PCR assays as the results are greatly influenced by the nucleic acid quality [12, 13]. As the conventional manual nucleic acid extraction methods are prone to contamination and inter- and intraoperator variability [14, 15], various automated nucleic acid extraction methods have been introduced.

Some automated nucleic acid extraction systems including the easyMGA system (Biomérieux), the Qiasymphony (Qiagen), Biorobot EZ1, and MgaNA pure Compact (Roche) were evaluated for stool, nasal, and nasopharyngeal aspirate samples in previous study [16–18]. However, these automated nucleic acid extraction systems were evaluated using multiplex PCR or multiples real-time PCR assays which are capable of detecting only up to 5 viruses simultaneously in a single patient sample. Only recently, multiple real-time PCR assay which is capable detecting more than 14 viruses simultaneously was developed and widely used [19, 20]. But a comprehensive comparison of the automated extraction systems for these multiplex RT-PCR using nasopharyngeal aspirate samples has never been carried out. The EZ1 Advanced XL and the QIAcube systems have been popularly used in medical laboratories for DNA/RNA extraction, whereas the MICROLAB Nimbus IVD system is newly introduced. Therefore, we evaluated the three different automated nucleic acid extraction systems for multiplex RT-PCR using clinical nasopharyngeal swab specimens.

## 2. Materials and Methods

### 2.1. Clinical Specimen Collection

A total of 84 nasopharyngeal swabs (Universal Transport Medium, Copan Diagnostics, Murrieta, CA, USA) were collected from 20 adult and 64 pediatric patients with signs and/or symptoms of the respiratory infection between February and July, 2012. All specimens were stored at 2–8°C for up to 72 hours prior to processing. The study was approved by the Institutional Review Boards of Gangnam Severance Hospital.

### 2.2. Nucleic Acid Extraction

Three different automated systems were used for the nucleic acid extraction. QIAcube system (Qiagen, Hilden, Germany) with QIAamp MinElute Virus Spin Kit (Qiagen), EZ1 Advanced XL system (Qiagen) with EZ1 Advanced XL Virus Mini kit v2.0 (Qiagen), and MICROLAB Nimbus IVD system (Hamilton, Reno, NV, USA) with STARMag96 Virus kit (Seegene, Seoul, Korea) were evaluated. Three aliquot samples were separated from each of the specimens, and nucleic acid of each aliquot samples was extracted on the same day by three different automated systems in a single laboratory. The sample and elution volumes used in this study were 150 *μ*L and 60 *μ*L for the QIAcube system, 400 *μ*L and 60 *μ*L for the EZ1 Advanced XL system, and 600 *μ*L and 100 *μ*L for the MICROLAB Nimbus IVD system, respectively. For the quality control of the entire nucleic acid extraction process and RT-PCR, 10 *μ*L of bacteriophage MS2 (AnyplexTMII RV16 detection, Seegene) was added to each sample as the internal control in order to check the entire process from nucleic acid extraction to PCR. cDNA was synthesized using the cDNA Synthesis Premix (Seegene) which included reverse transcriptase and a random hexamer, according to the manufacturer's protocol.

### 2.3. Real-Time PCR and Melting Curve Analysis

Anyplex II RV16 Detection kit (Seegene) was used to detect 14 RNA viruses and 2 DNA viruses including human adenovirus (ADV), influenza A and B viruses (FluA, FluB), human parainfluenza viruses 1/2/3/4 (PIV1/2/3/4), human rhinovirus A/B/C (RV A/B/C), human respiratory syncytial viruses A and B (RSV-A, RSV-B), human bocaviruses 1/2/3/4 (BoV1/2/3/4), human coronaviruses 229E, NL63 and OC43 (CoV-229E, CoV-NL63, CoV-OC43), human metapneumovirus (MPV), and human enterovirus (EV). A real-time PCR reaction mixture was prepared with as follows: 8 *μ*L cDNA, 4 *μ*L 5x RV primer, 4 *μ*L 8-methoxypsoralen solution, and 4 *μ*L 5x Master Mix. Seegene's unique real-time PCR and melting curve analysis technique called “Tagging Oligonucleotide Cleavage and Extension” assay (http://www.seegene.co.kr/neo/en/introduction/core_toce.php) was performed using CFX96 real-time PCR detection system (Bio-Rad, Hercules, CA, USA) as follows: (1) 1 cycle of initial denaturation at 95°C for 15 min, (2) 50 cycles of denaturation at 94°C for 30 sec, annealing at 60°C for 1 min, and extension at 72°C for 30 sec, (3) 1 cycle of cooling the reaction mixture at 55°C for 30 sec, and (4) melting double-stranded DNA into single strands by raising the temperature to 85°C. The fluorescence was measured continuously during the temperature rise from 55°C. And, the melting peaks were derived from the initial fluorescence (*F*) versus temperature (*T*) curves by plotting the negative derivative of fluorescence over temperature versus temperature (−*dF*/*dT*  versus  *T*) by Seegene software [19]. If internal control was not detected in real-time PCR reaction, the results were sorted as “invalid results” by Seegene software. Plasmids containing the target sequence were included as positive controls.

### 2.4. Analytical Sensitivity of the Automated Nucleic Acid Extraction Systems

One DNA (ADV) and 2 RNA (RSV-A and FluA) viral reference strains (ATCC VR-3, ADV; ATCC VR-26, RSV-A; ATCC VR-544, FluA) were obtained from the Korean Bank for Pathogenic Viruses (Korea University College of Medicine, Seoul, Republic of Korea). The reference strains were serially diluted with 10-fold saline buffer and they were made as five-level samples: ADV (10^−6^ to 10^−10^), FluA (10^−5^ to 10^−9^), and RSV (10^−4^ to 10^−8^). And each diluted sample was extracted 5 times using the 3 different automated nucleic acid extraction systems.

### 2.5. Interpretation of the Results

When all 2 or 3 of the automated nucleic acid extraction systems yielded the same results, they were considered as a true positive and we considered it as a true negative when all 3 of the automated nucleic acid extraction systems yielded “not detected.” In case of positive results from only 1 of the three automated nucleic acid extraction systems, the confirmatory test performed with the Seeplex RV15 ACE Detection kit (Seegene). When the confirmatory test also yielded negative result, it was considered as a false positive [16]. Among these positive results from only 1 of the three automated nucleic acid extraction systems, three pathogens (FluB, HRV, and HBoV) were detected when repeated with the Seeplex RV15 ACE Detection kit (Seegene). These discordant results were also confirmed by PCR and sequencing. The following primers were used for the sequencing analysis: FluB-NF (GTC CAT CAA GCT CCA GTT TT), FluB-NR (TCT TCT TAC AGC TTG CTT GC) (145 bp), HRV-5′NCRF (GCA CTT CTG TTT CCC C), HRV-5′NCRR (CGG ACA CCC AAA GTA G) (380 bp), HBoV-NP1F (GAC CTC TGT AAG TAC TAT TAC), HBoV-NP1R (CTC TGT GTT GAC TGA ATA CAG) (354 bp) [21].

### 2.6. Statistical Analysis

Statistical analysis was performed using SPSS Statistics 20.0 (SPSS Inc., Chicago, IL, USA) and Analyse-it 2.22 (Analyse-it Software Ltd., Leeds, UK). Kappa coefficients were calculated to estimate the agreement between the results by different utilized methods.

## 3. Results

### 3.1. Detection of Respiratory Viruses in Clinical Specimens

Among the 84 nasopharyngeal swab specimens, viral pathogens were detected in 44 specimens (detection rate: 52.4%). A total of 58 pathogens including multiple infections were detected in 44 specimens and the proportions of single and dual and triple infections were 75.0%, 18.2% and 6.8%, respectively. Based on frequency of detection, RV (32.8%) was more than one-third of detected pathogens, followed by MPV (13.8%), PIV3 (13.8%), RSV-A (12.1%), and ADV (8.6%). And RV was the predominant pathogen in case of both single (30.3%, *n* = 10/19) and dual (87.5%, *n* = 7/8) respiratory infections (Table 1).

### 3.2. Comparison of the PCR Results from 3 Automated Nucleic Acid Extraction Systems

The numbers of positives for respiratory viruses ranged from 54 to 59. The nucleic acids extracted by EZ1 Advanced XL showed higher positive rates for virus detection than the others. Among the discrepant results, three positive nucleic acid extracts, which were obtained by only one of the three automated nucleic acid extraction systems, were confirmed by sequencing. Two positive nucleic acid extracts including RV (*n* = 1) and BoV (*n* = 1) were obtained by the QIAcube system, while one FluB positive nucleic acid extract was obtained with EZ1 Advanced XL (Table 2). On the basis of Section 2.5, sensitivity, specificity, concordance rate, and kappa coefficient for the 3 automated nucleic acid extraction systems are shown in Table 3. The percent sensitivity and specificity of the 3 systems ranged from 87.2% to 93.3% and from 82.9% to 94.6%, respectively. The concordance rates between the true results and the 3 systems ranged from 88.3% to 94.2%. The kappa coefficients ranged from 0.76 to 0.88 (*P* < 0.001 for all values). The QIAcube system yielded the best percent sensitivity and concordance rate, while the MICROLAB Nimbus IVD system demonstrated the lowest sensitivity and the highest specificity. Sensitivity and specificity of the 3 systems were also evaluated for the most commonly detected 5 viral pathogens. The QIAcube system showed the highest sensitivity for the RNA viruses (RV, MPV, PIV3, and RSV-A), while the EZ1 Advanced XL system demonstrated the best percent sensitivity for the ADV.

One of two BoVs was not detected by the EZ1 Advanced XL system, while all three were detected by the QIAcube system (Table 2). For DNA viruses, including ADV and BoV, the QIAcube system and the EZ1 Advanced XL system demonstrated equal sensitivity of 85.7%.

### 3.3. Characteristics of the Three Automated Nucleic Acid Extraction Systems

Basic characteristics of the 3 automated nucleic acid extraction systems are summarized in Table 3. The QIAcube system employs the spin column principle, while the EZ1 Advanced XL and the MICROLAB Nimbus IVD systems use magnetic particles for the nucleic acid extraction. While sample and elution volumes can be adjusted in the QIAcube and the EZ1 Advanced XL systems, they are fixed in the MICROLAB Nimbus IVD system. The EZ1 Advanced XL system demonstrated the shortest turnaround time (TAT) per sample, followed by the QIAcube system and the MICROLAB Nimbus IVD system. The MICROLAB Nimbus IVD system was suitable for the downstream applications as it had the highest sample capacity and the automated PCR setup function.

### 3.4. Analytical Sensitivity of the Three Automated Nucleic Acid Extraction Systems

The analytical sensitivity of the 3 automated nucleic acid extraction systems is showed in Table 4. The EZ1 Advanced XL system demonstrated the best analytical sensitivity for all 3 of the viral strains. There was little difference between the QIAcube and the MICROLAB Nimbus IVD systems with regards to analytical sensitivity.

## 4. Discussion

If a specimen with positive results for the same virus by more than 2 of 3 systems was used as the “gold standard,” the performance of the QIAcube system was superior to the EZ1 Advanced XL system and the MICROLAB Nimbus IVD system in sensitivity and concordance rate (Table 3). The EZ1 Advanced XL system showed relatively high number of the discrepant results (*n* = 12) and false positives (*n* = 7), especially for ADV (3 false positives). However, it showed the best analytical sensitivity for ADV-3, FluA, and RSV-A (Table 4) and as well as the highest sensitivity for ADV in clinical specimens (Table 3). As the analytical sensitivity of the confirming test including the Seeplex RV15 ACE Detection kit (Seegene) and sequencing analysis could be lower than Anyplex II RV16 Detection kit (Seegene) [22], some of these false positive results could include true positive results. Therefore, we discerned that the EZ1 Advanced XL system is more suitable for ADV virus detection. The MICROLAB Nimbus IVD system showed the highest specificity (94.6%, only two false positives), but it demonstrated the lowest sensitivity (87.2%) with 6 false negatives (Table 3).

The sample and elution volumes used in this study were 150 *μ*L and 60 *μ*L for the QIAcube system, 400 *μ*L and 60 *μ*L for the EZ1 Advanced XL system, and 600 *μ*L and 100 *μ*L for the MICROLAB Nimbus IVD system, respectively. The relative concentration ratios of sample volume to elution volume were 2.5 for the QIAcube system, 6.7 for the EZ1 Advanced XL system, and 6.0 for the MICROLAB Nimbus IVD system, respectively. We used QIAcube system with sample volume 150 *μ*L and elution volume 60 *μ*L routinely in our laboratory. Therefore, we adjusted the sample volume and elution volume in the MICROLAB Nimbus IVD system and the EZ1 Advanced XL system similarly, but we could not do this in QIAcube system. Although the QIAcube system was performed with the lowest sample/elution volume ratio, it showed the best performance.

Previously, Chan et al. evaluated the performance of the NucliSens easyMAG (bioMerieux, Marcy l'Etoile, France) and BioRot 9604 automated nucleic acid extraction systems (Qiagen) in comparison with the manual QIAamp extraction method (Qiagen). In their study, these three different methods were evaluated with different sample volumes and elution volumes. The relative concentration ratios of the sample volume to elution volume were 4.5 for the NucliSens easyMAG, 2.5 for the BioRot 9604, and 1.0–2.3 for the manual QIAamp, respectively. However, the nucleic acids obtained by all three methods gave comparable sensitivities in PCR tests, and the three methods gave comparable viral loads [16] Therefore, we thought that the relative concentrative effect of the eluted nucleic acid may have little effect on the results of RT-PCR based methods. In a previous study, the MagNA Pure Compact machine with the MagNAPure Compact Nucleic Acid Isolation Kit I (Roche, Indianapolis, IN, USA) demonstrated more than 4-fold higher DNA recovery from the* S. pyogenes *than the other automated extraction systems including KingFisher-ML (ThermoFisher Scientific Inc., Worcester, MA, USA), Biorobot EZ1 (Qiagen), easyMAG (bioMerieux), and Biorobot MDX (Qiagen); however, it was less efficient in RNA purification from RSV and influenza A virus viruses. This may be due to RNA degradation or inefficient RNA binding to the magnetic beads [17]. Actually, the MagNAPure Compact Nucleic Acid Isolation Kit I was preferred for DNA extraction, although it did not include carried RNA. Inclusion of carried RNA could enhance the yield of a very few target molecules and reduce the chances of viral RNA degradation. In our study, all three extraction kits included carried RNA, and all three systems showed comparable efficiencies of RNA and DNA extraction (Table 4).

Turnaround time (TAT) per sample of the QIAcube system, EZ1 Advanced XL, and the MICROLAB Nimbus IVD systems were 7.5 minutes, 3.2 minutes, and 3.1 minutes, respectively. Nucleic acid extraction systems using the magnetic particle principle had the shorter TAT per sample than the system which employs the spin column principle, probably due to its simplicity to perform [23].

Therefore, the systems using the magnetic particle may be more preferred for more rapid identification of the viral pathogens. The longer hands-on time of the MICROLAB Nimbus IVD systems was drawback, but it has the capability to run 3-4 fold more samples than other comparison systems at the same time and this system integrates fully automated steps from nucleic extraction to PCR setup function, allowing human errors to be minimized and providing more reliable results. This system is more suitable for laboratory which was carried on large samples for PCR.

## 5. Conclusion 

In the present study, the QIAcube system showed the fewest false negative results and the best concordance rate, and it may be more suitable for detecting various viruses, including RNA and DNA virus strains. However, each system demonstrated different sensitivity and specificity for the detection of certain viral pathogens and different characteristics such as the carrier RNA, TAT, sample capacity, and automated PCR setup function. Therefore, according to the characteristics of the target patient group and the laboratory, these factors should be considered when the new nucleic acid extraction system is introduced into the laboratory.

## Figures and Tables

**Table 1:** Distribution of respiratory viruses in 44 positive nasopharyngeal swab specimens.

Virus type	Single infection (*n* = 33)	Dual infection (*n* = 8)	Triple infection^a^ (*n* = 3)	Total (%) (*n* = 44)
RV	10	7	2	19 (32.8)
MPV	3	2	3	8 (13.8)
PIV3	5	2	1	8 (13.8)
RSV-A	6	1	0	7 (12.1)
ADV	1	2	2	5 (8.6)
FluB	2	0	1	3 (5.2)
FluA	2	0	0	2 (3.4)
BoV	1	1	0	2 (3.4)
EV	1	1	0	2 (3.4)
CoV-OC43	1	0	0	1 (1.7)
PIV1	1	0	0	1 (1.7)
Total (%)	**33 (56.9)**	**16 (27.6)**	**9 (15.5)**	**58 (100.0)**

^a^RV + MPV + ADV (*n* = 1), RV + MPV + FluB (*n* = 1) and MPV + ADV + PIV3 (*n* = 1).

RV: human rhinovirus; MPV: human metapneumovirus; PIV3: human parainfluenza virus 3; RSV-A: human respiratory syncytial virus A; ADV: human adenovirus; FluB: influenza B; FluA: influenza A; BoV: human bocavirus; EV: human enterovirus; CoV-OC43: human coronavirus OC43; PIV1: human metapneumovirus.

**Table 2:** Comparison of detection of respiratory virus by three automatic extraction methods from nasopharyngeal swab specimens.

Virus	QIAcube	EZ1 advanced XL	MICROLAB Nimbus IVD	Total number of specimens
RV	+	+	+	14
	+	−	+	3
	+	−	−	1^a^
	−	+	+	1
	−	+	−	2^b^

MPV	+	+	+	7
	+	+	−	1

PIV3	+	+	+	8

PIV1	+	+	+	1
	−	+	−	1^b^

PIV4	+	−	−	1^b^

RSV-A	+	+	+	7
	−	+	−	1^b^

ADV	+	+	+	2
	−	+	+	1
	+	+	−	2
	−	+	−	3^b^

FluB	+	+	+	2
	−	+	−	1^a^

FluA	+	+	+	2

BoV	+	+	+	1
	+	−	−	1^a^

EV	+	+	+	2
	+	−	−	1^b^

CoV-OC43	1	1	1	1

CoV-NL63	−	−	+	1^b^

CoV-229E	−	−	+	1^b^

Total	**57**	**59**	**54**	**68**

^a^Virus detected by only 1 of the three automated nucleic acid extraction systems, and we confirmed this result by sequencing analysis and confirmed it as “true positive.”

^b^Virus detected by only 1 of the three automated nucleic acid extraction systems, but they were not detected by repeated test with the Seeplex RV15 ACE Detection kit (Seegene).

**Table 3:** Comparison of the PCR results from 3 automated nucleic acid extraction systems using a total of 84 nasopharyngeal specimens.

	QIAcube	EZ1 advanced XL	MICROLAB Nimbus IVD
Positive results	45 (53.6%)	45 (53.6%)	43 (51.2%)
True positive^a^	42	38	41
False positive^a^ (virus type)	3 (PIV4, EV, RSV-B)	7 (PIV1, ADV (*n* = 3), RSV-A, RV (*n* = 2))	2 (CoV-NL63, CoV-229E)
Negative results	39 (46.4%)	39 (46.4%)	41 (48.8%)
True negative^a^	36	34	35
False negative^a^ (virus type)	3 (FluB, RV, ADV)	5 (BoV, RV (*n* = 4))	6 (FluB, RV, MPV, BoV, ADV (*n* = 2))
Sensitivity (%)	93.3	88.4	87.2
Specificity (%)	92.3	82.9	94.6
Concordance rate (%)	94.2	88.3	92.2
Kappa coefficient^b^ (95% CI)	0.88 (0.79 to 0.97)	0.76 (0.64 to 0.89)	0.84 (0.74 to 0.95)
RV sensitivity/specificity	94.7/100.0	78.9/96.9	94.7/100.0
MPV sensitivity/specificity	100.0/100.0	100.0/100.0	85.0/100.0
PIV3 sensitivity/specificity	100.0/100.0	100.0/100.0	100.0/100.0
RSV-A sensitivity/specificity	100.0/100.0	100.0/98.7	100.0/100.0
ADV sensitivity/specificity	80.0/100.0	100.0/96.2	60.0/100.0
Characteristics of three systems			
Principle	Spin column	Magnetic particle	Magnetic particle
Sample volume (*μ*L)	≤200	100–400	600
Elution volume (*μ*L)	20–150	60–150	100
Turnaround time^c^ (min)	90	45	150
Sample capacity	12	14	48
Automated PCR set-up	Impossible	Impossible	Possible

^a^When all three automated nucleic acid extraction systems yielded the same results, they were considered “true positive” or “true negative.” If only 1 system yielded negative result, it was considered “false negative.” When the pathogen was detected from only 1 system, multiplex PCR and sequencing analysis were performed to confirm the result. ^b^ *P* < 0.0001 for all values. ^c^includes lysis step but not the hands-on time.

**Table 4:** Analytical sensitivity of the three automated nucleic acid extraction systems.

Virus strain dilution factor	ADV-3 (detected/replicates)					FluA (detected/replicates)					RSV-A (detected/replicates)				
	10^−6^	10^−7^	10^−8^	10^−9^	10^−10^	10^−5^	10^−6^	10^−7^	10^−8^	10^−9^	10^−4^	10^−5^	10^−6^	10^−7^	10^−8^
QIAcube (QIAGEN)	5/5	3/5	0/5	0/5	0/5	5/5	2/5	1/5	0/5	0/5	5/5	4/5	0/5	0/5	0/5
EZ1 (QIAGEN)	5/5	5/5	0/5	0/5	0/5	5/5	5/5	2/5	1/5	0/5	5/5	5/5	3/5	1/5	0/5
NIMBUS (Seegene)	5/5	4/5	0/5	0/5	0/5	5/5	4/5	0/5	0/5	0/5	5/5	2/5	1/5	0/5	0/5
